# Expression Profile of the *Schistosoma japonicum* Degradome Reveals Differential Protease Expression Patterns and Potential Anti-schistosomal Intervention Targets

**DOI:** 10.1371/journal.pcbi.1003856

**Published:** 2014-10-02

**Authors:** Shuai Liu, Pengfei Cai, Xianyu Piao, Nan Hou, Xiaosu Zhou, Chuang Wu, Heng Wang, Qijun Chen

**Affiliations:** 1 MOH Key Laboratory of Systems Biology of Pathogens, Institute of Pathogen Biology, Chinese Academy of Medical Sciences and Peking Union Medical College, Beijing, China; 2 Key Laboratory of Zoonosis, Jilin University, Changchun, China; 3 Department of Microbiology and Parasitology, Institute of Basic Medicine, Chinese Academy of Medical Sciences and Peking Union Medical College, Beijing, China; University of North Carolina at Charlotte, United States of America

## Abstract

Blood fluke proteases play pivotal roles in the processes of invasion, nutrition acquisition, immune evasion, and other host-parasite interactions. Hundreds of genes encoding putative proteases have been identified in the recently published schistosome genomes. However, the expression profiles of these proteases in *Schistosoma* species have not yet been systematically analyzed. We retrieved and culled the redundant protease sequences of *Schistosoma japonicum*, *Schistosoma mansoni*, *Echinococcus multilocularis*, and *Clonorchis sinensis* from public databases utilizing bioinformatic approaches. The degradomes of the four parasitic organisms and *Homo sapiens* were then comparatively analyzed. A total of 262 *S. japonicum* protease sequences were obtained and the expression profiles generated using whole-genome microarray. Four main clusters of protease genes with different expression patterns were identified: proteases up-regulated in hepatic schistosomula and adult worms, egg-specific or predominantly expressed proteases, cercaria-specific or predominantly expressed proteases, and constantly expressed proteases. A subset of protease genes with different expression patterns were further validated using real-time quantitative PCR. The present study represents the most comprehensive analysis of a degradome in *Schistosoma* species to date. These results provide a firm foundation for future research on the specific function(s) of individual proteases and may help to refine anti-proteolytic strategies in blood flukes.

## Author Summary

Parasite proteases play critical roles in host-parasite interactions and thus are considered to be potential anti-schistosomal targets. Although numerous schistosome proteases have been predicted based on recently published genomes, no systematic analysis of their expression in *Schistosoma* species has been performed. Thus, we comparatively analyzed the degradomes of four parasitic organisms and human host, and performed whole-genome microarray analysis to analyze the expression profile of the *Schistosoma japonicum* degradome at four developmental stages. The expression profile generated for the *S. japonicum* degradome was divided into four main clusters with different expression patterns, and a subset of selected proteases were further validated using real-time quantitative PCR. Our work is the most comprehensive analysis of a degradome in *Schistosoma* species to date. Many protease genes were first characterized in blood flukes, and some could be treated as potential anti-schistosomal targets for intensive research in the future. The results provide a firm foundation for deep study on the specific function(s) of individual proteases or protease families in schistosomes.

## Introduction

Schistosomiasis is one of the most socioeconomic parasitic diseases, afflicting millions of people in many tropical and subtropical countries [1]. Praziquantel is the only specific remedy for the disease and no effective vaccine is available [2]. However, long-term, repeated mass chemotherapy in endemic regions may give rise to praziquantel resistance, and *Schistosoma mansoni* strains resistant or insensitive to the drug have been found in some endemic areas [2]–[4]. Therefore, additional chemotherapeutic agents against blood flukes are desperately needed.

Proteases are considered druggable targets from the medical and chemical viewpoints because of their known enzymatic mechanism and regulatory roles in many pathologies [5]. A number of protease inhibitors have been developed and approved for the treatment of various human diseases [6]. Parasite proteases contribute to pathogenesis in a variety of ways, including invasion, nutrition acquisition, immune evasion, and other host-parasite interactions [7]. Although most parasite-derived proteases have counterparts in their mammalian hosts, the mammalian proteases are often sequestered in different cell organelles or play distinct biological roles [8]. For example, cysteine proteases perform critical functions in extracellular proteolysis in parasites, but their mammalian counterparts are found predominantly in intracellular organelles. These discoveries make proteases promising targets for the development of novel immunological or chemotherapeutic anti-parasite agents [9]. Indeed, cysteine protease inhibitor K11777 has been tested in the murine model of schistosomiasis, and the remarkable reduction in worm burden and pathology validated schistosome cysteine proteases as novel potential drug targets for chemotherapy [10], [11].

Recently, draft genome sequences were published for the three major pathogens of human schistosomiasis: *Schistosoma japonicum*, *S. mansoni*, and *Schistosoma haematobium* [12]–[14]. These genomic resources have provided insight into the molecular basis of schistosome biology, host-parasite interactions, and the pathogenesis of schistosomiasis, which will facilitate innovations in schistosomiasis control [15]. The degradome is defined as the complete set of proteases present in an organism [16]. The recent availability of whole genomic sequences from blood flukes has led us to predict the contents of the degradomes in *Schistosoma* species. In *S. japonicum*, 314 putative proteases have been identified that can be divided into five major classes of proteases: aspartic, cysteine, metallo-, serine, and threonine [12]. The vast majority of schistosome proteases have been identified using predicted proteomes, and only a few have been functionally characterized. Choosing a putative protease gene for further investigation will be difficult without transcript information. Because schistosome parasites have a complicated developmental lifecycle comprising seven morphologically discrete stages, and the protease genes may be expressed in different lifecycle stages. Four developmental stages are closely associated with mammalian hosts: cercariae, by which mammalian hosts are infected; juvenile schistosomula, which enter mammalian hosts' capillaries and lymphatic vessels en route to the lungs and liver; adult worms, which migrate to the veins of the intestines or bladder and produce eggs; and eggs, which cause serious granulomatous reactions and fibrosis in the affected organs [17]. Elucidation of the expression of the proteases in these four important stages of the parasite will contribute to the future function dissection of the enzymes, which will facilitate discovery of anti-schistosomal intervention targets.

However, no systematic analysis of degradome profiles has been performed in *Schistosoma* species to date. Therefore, we used whole-genome microarray analysis to profile the expression of the majority of protease genes in these four developmental stages of *S. japonicum*. The gene expression patterns of a subset of proteases were further validated using real-time quantitative PCR (qRT-PCR). The results obtained from this work provide a foundation for the further functional characterization of protease genes in *Schistosoma* species.

## Materials and Methods

### Ethical statement

All procedures performed on animals within this study were conducted following animal husbandry guidelines of the Chinese Academy of Medical Sciences and with permission from the Experimental Animal Committee with the Ethical Clearance Number IPB-2011-6.

### Protease sequence retrieval and analysis

The degradomes of four parasitic organisms with known genome sequences were analyzed in this study. A total of 314 *S. japonicum* protease sequences predicted in genome-wide [12], and 253 sequences obtained from the MEROPS database [18] were integrated to generate the degradome of *S. japonicum*. The degradome of *S. mansoni* was composed of protease sequences predicted according to the putative proteome [13]. The degradome of *Clonorchis sinensis* comprised protease sequences from the *MEROPS* database. CD-HIT v4.5.4 software (http://www.bioinformatics.org/cd-hit/) was used to remove redundant sequences, with the standard of 90% identity and 80% coverage between two sequences (the shorter one was eliminated). If the identity was 100% between two sequences with more than 100 aligned consecutive amino acid residues, one sequence was eliminated manually.

Next, the degradome of *S. japonicum* was analyzed using several bioinformatic approaches. Protease sequences were functionally annotated using Blast2GO [19], and the output provided as combined graphics in three categories of gene ontology (GO) terms: biological processes, molecular functions, and cellular components. The KEGG automated annotation server (KAAS) was used to assign pathway-based functional orthology to protease sequences [20]. Signal peptides were predicted using the SignalP 4.1 server [21], and transmembrane helices were predicted using TMHMM 2.0 [22].

### Phylogenetic analysis of the schistosome cathepsin gene family

The *S. japonicum* degradome sequences were searched to identify cathepsin proteins using BLASTp program with the published schistosome cathepsin protein sequences and *Homo sapiens* cathepsin Aprotein sequence (4CI9_A) as query sequences. All obtained protein sequences were further examined for the presence of cathepsin conserved motif and domains by searching the Conserved Domain Database (v. 3. 11) on NCBI [23], [24]. The amino acid sequences of cathepsins from *S. japonicum* and *S. mansoni* were first aligned using ClustalX [25], and then refined manually. Finally, phylogenetic tree was constructed using MEGA 5.0 software by the neighbor-joining (NJ) method, and the bootstrap test was replicated 1000 times [26].

### Parasite materials

The freshly released cercariae were harvested from *S. japonicum*-infected *Oncomelania hupensis* provided by Hunan Institute of Parasitic Diseases, Yueyang, China. Hepatic schistosomula were isolated from infected New Zealand rabbits at 2 weeks post-infection. Mixed adult worms were isolated from infected rabbits at 6 weeks post-infection. Male and female worms were manually separated with the aid of a light microscope. Eggs were purified from liver tissues of infected rabbits by enzyme digestion method [27]. All parasites were soaked in RNAlater solution (Ambion, CA, USA), and stored at −80°C until total RNA was isolated.

### Total RNA isolation

Total RNAs were isolated from parasites at different developmental stages (eggs, cercariae, hepatic schistosomula, and adult worms) using RNeasy Mini kit (QIAGEN), and the contaminating genomic DNA were removed from RNA samples with Tubro DNA-free kit (Ambion, CA, USA). The quantity and quality of the RNA samples were assessed by NanoDropND-1000 spectrophotometer (NanoDrop Technologies, Wilmington, DE) and denaturing agarose gel electrophoresis.

### Microarray analysis of *S. japonicum* degradome

Schistosome genome-wide microarray was used to analysis the expression profile of the *S. japonicum* degradome. The design and construction of the microarray, and the methods used in microarray hybridization and feature extraction have been previously reported [28]. Microarray hybridization was performed in three biological replicates for all samples. Raw data and normalized gene level data from the array have been deposited at the public database Gene Expression Omnibus (http://www.ncbi.nlm.nih.gov/geo) under accession numbers for the platform GPL18617, and series GSE57143. Finally, local BLAST searches were performed to identify the microarray sequences corresponding to *S. japonicum* protease sequences which were used as query sequences. For protease sequences with more than one microarray sequences, the highest expression value was considered. Protease genes were considered as statistical differentially expressed by expression fold-change ≥2 between any two compared developmental stages, and *p*-value <0.05 (one tailed Student's *t*-test). The coefficient of variation (CV) was employed to extract the constantly expressed protease genes among the four developmental stages by cut-off value of 0.15. Hierarchical clustering analysis of selected genes was performed to generated heat maps using Cluster 3.0 software [29], and Heatmap Builder 1.0 software [30].

### Real-time quantitative PCR

A subset of protease genes with different expression patterns were selected for further validation using qRT-PCR as previously described [28]. Reactions were carried out in technological triplicate on the 7300 Real-Time PCR system (Applied Biosystems) using Brilliant II SYBR Green QPCR Master Mix (Agilent Technologies, USA) according to the manufacturer's instructions. 26S proteasome non-ATPase regulatory subunit 4 (PSMD4), which has been validated as a reliable reference gene in transcriptomic analysis of *S. japonicum* [28], was employed as a control gene in the qRT-PCR analysis. The qRT-PCR primers were designed using Primer Express 3.0 software (Applied Biosystems, Foster City, USA) (Table S1). The relative expression level of each gene was analyzed using the software SDS 1.4 (Applied Biosystems).

## Results and Discussion

### Overview of the *S. japonicum* degradome

After culling the redundant sequences, we identified a total of 262 genes encoding known or putative proteases among 64 families (Table 1 and S2), comprising approximately 2% of the *S. japonicum* predicted proteome. Depending on the key residue for protease catalytic mechanism, all of these proteases can be grouped into five catalytic classes: aspartyl, cysteine, metallo-, serine, and threonine. In this study, we culled the redundant protease sequences according to a strict standard. For instance, short sequences were removed under conditions of 90% identity and 80% coverage between two sequences. Indeed, we found that some short predicted protein sequences aligned to the same long protein sequence with a complete coding sequence on NCBI. Therefore, the number of proteases we identified is less than the number of proteases (314 protease sequences) predicted from the recently published genome sequence of *S. japonicum*. Similarly, a total of 255 putative protease sequences (269 proteases in our result) were identified using different data mining methodologies, compared to 335 protease sequences identified from proteins predicted for the *S. mansoni* genome [31]. Most protease families have shrunk, and the greatest reduction in number was the C01 family, which is now at half of previously published numbers (16 sequences vs. 31 sequences). However, our data added several new protease families for *S. japonicum*, including C83, C85, C86, and C97.

**Table 1:** General information about *S. japonicum* degradome.

Protease class	Num. protease sequences	Num. protease families	Proteases with predicted transmembrane helices	Proteases with predicted signal peptide
Aspartic	13	5	4	3
Cysteine	77	21	2	16
Metallo	101	22	17	9
Serine	54	13	14	14
Threonine	17	3	1	1
Total	262	64	38	43

### Comparative analysis of the degradomes in parasitic flatworms and human host

**Figure 1:**
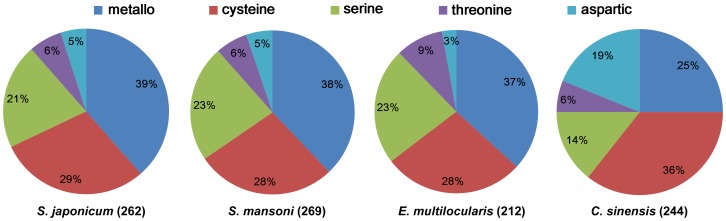
Proportions of each protease class in the degradomes of four parasites. *S. japonicum* (262 proteases), *S. mansoni* (269 proteases), *E. multilocularis* (212 proteases), and C. sinensis (244 proteases).

Genome sequencing has paved the way for a systematic dissection of parasite biology, and an increasing number of parasite genomes have been decoded recently [15], [32]–[35]. Here, we chose three more parasitic worms for comparative analysis of their degradomes: *S. mansoni* (Platyhelminthes, Trematoda), *C. sinensis* (Platyhelminthes, Trematoda), and *Echinococcus multilocularis* (Platyhelminthes, Cestoda). After eliminating the redundant sequences, the total numbers of known or putative proteases in the four parasite species were as follows: 262 (*S. japonicum*), 269 (*S. mansoni*), 212 (*E. multilocularis*, the lists were abstracted from the genome, supplementary table 13.9 [33]) and 244 (*C. sinensis*) (See more details in Table S3). Except *C. sinensis*, the proportion of protease classes in each of the parasites was largely in harmony with each other (Figure 1). A significant expansion was observed in the relative proportion of aspartic proteases in *C. sinensis* compared to that of the other three species, which was mainly due to A01 family (41 members of the A01 family in *C. sinensis*, compared to five genes in *S. japonicum*, seven in *S. mansoni*, and only one in *E. multilocularis*). On the contrary, the number of serine proteases and metalloproteases was obviously reduced in *C. sinensis* (the first was S09 family of serine proteases and M08 family of metalloproteases). Although the proportion of protease classes was largely similar between *Schistosoma* species and *E. multilocularis*, there were several protease families exhibiting significant difference, such as C01, M08 and M13 family (See more details in Table S3).

We further performed comparative analysis of the degradomes between parasitic flatworms and human host (the degradome of human was abstracted from the mammalian degradome database [16]). There are 54 common protease families among the four flatworms, of which 53 families are shared between flatworm and human. Notably, one protease family (C83) is obviously exclusive to these four parasitic flatworms and may be ideal anti-parasite targets for the future investigation. Moreover, we performed BLASTp searches against the non-redundant protein sequences (nr) database of *S. mansoni* (taxid: 6183) and *H. sapiens* (taxid: 9606) on NCBI using the *S. japonicum* protease sequences as query sequences. The results indicated that the majority of schistosome proteases shared relatively low sequence identity with their homologous counterparts in *H. sapiens*. More importantly, some crystal structures of these human proteases have been resolved, which lays good foundation for the future screening of compounds selectively inhibiting the parasite proteases based on structural disparity (Table S2). Meanwhile, there were several schistosome proteases sharing relatively high sequence identity with their counterparts in *H. sapiens*, such as O-sialoglycoprotein endopeptidase (Sjp_0083850, M22 family), methionine aminopeptidase 2 (ACU78097.1, M24 family) and 20S proteasome subunits (T01 family) (Table S2). It might suggest that these proteases evolve slowly at the sequence level, and still retain their functions. In addition, we found that a few long *S. japonicum* protease sequences aligned with more than one short non-redundant *S. mansoni* protease sequence in different sequence positions, and vice versa. For example, the *S. japonicum* protease sequence (AAW26282.1) aligned with the *S. mansoni* protease sequence (Smp_061510.1) from the first amino acid to amino acid 570 (identity 83%), and the protease sequence (Smp_155220) from amino acid 579 to the last amino acid (identity 84%) (Figure S1). Thus, some short schistosome protease sequences may belong to the same protease sequence, considering the majority of proteases were predicted protein sequences based on schistosome genome sequences. The comparative analysis of sequences in the two *Schistosoma* species will further improve the quality of genomes and proteomes in the future [36].

### Gene ontology analysis of the *S. japonicum* degradome

**Figure 2:**
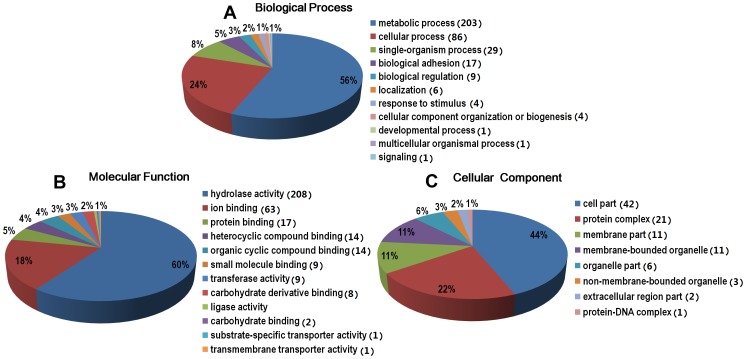
Gene ontology (GO) distributions for the *S. japonicum* degradome. The Blast2Go program defined the GO terms using three categories: (A) biological processes, (B) molecular functions, and (C) cellular component.

Gene ontology (GO) analysis was performed to summarize and explore the functional categories of the *S. japonicum* degradome in this study. A total of 240 of the 262 *S. japonicum* protease sequences were annotated with GO terms in three independent categories: biological processes (210 protease sequences), molecular functions (231 protease sequences), and cellular components (78 protease sequences) (Table S4). The biological processes analysis (level 2) showed that the predominant proteases (203, 56%) were involved in the response to metabolic processes (Figure 2A). In the case of molecular functions (level 3), the majority of proteases were annotated with hydrolase activity (208, 60%), which is in consistent with the molecular role of proteases in proteolysis (Figure 2B). Notably, proteases with the ion binding term (63, 18%) may be metal ion-dependent enzymes. Interestingly, only 78 proteases were annotated with GO terms in the cellular components analysis (level 3). Twenty-one proteases (22%) were components of protein complexes, including 14 proteases that were subunits of the proteasome core complex (Figure 2C, Table S4).

### Global expression profiles of the *S. japonicum* degradome at four developmental stages

**Figure 3:**
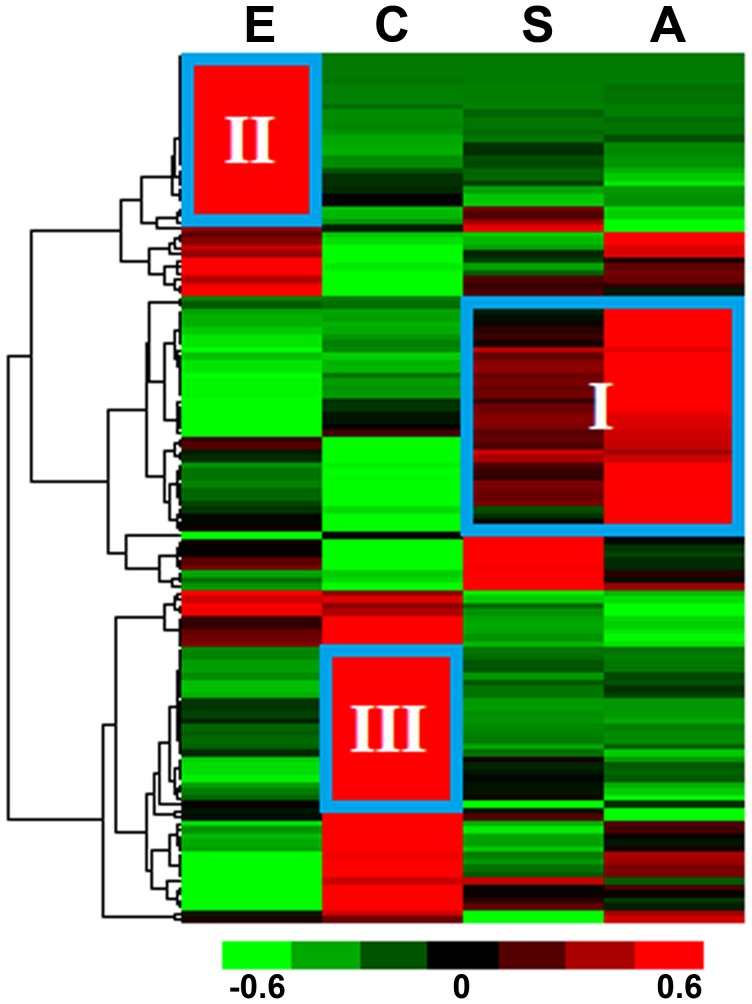
Hierarchical clustering of the expression profiles of the *S. japonicum* degradome at four developmental stages. The heat map of 136 differentially expressed protease genes extracted from the microarray dataset (E, eggs; C, cercariae; S, hepatic schistosomula; A, adult worm pairs). Three clusters of up-regulated protease genes were identified: I, genes significantly up-regulated in the schistosomula and adult stages; II, genes abundantly expressed in the egg stage; III, genes highly expressed in the cercaria stage. The color scale represents relative expression levels, with red as up-regulated, green as down-regulated, and black as unchanged.

**Figure 4:**
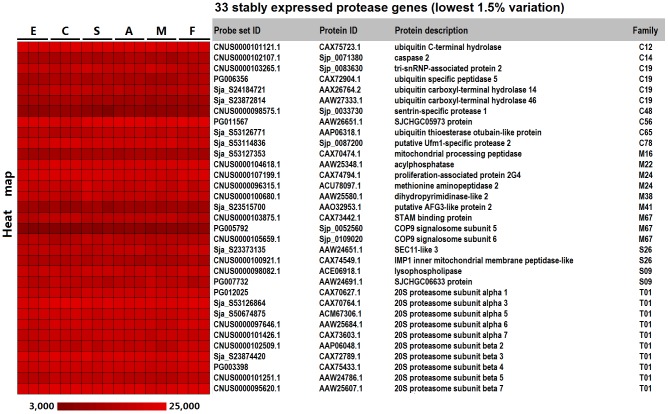
Constantlyexpressed *S. japonicum* protease genes in four developmental stages were identified by microarray analysis. The heat map shows the fluorescent intensity values for the 33protease genes with the lowest coefficient of variation (1.5%) among the four developmental stages (E, eggs; C, cercariae; S, hepatic schistosomula; A, adult worm pairs; M, adult male worms; F, adult female worms). Each of the stages contained three biological replicates.

**Figure 5:**
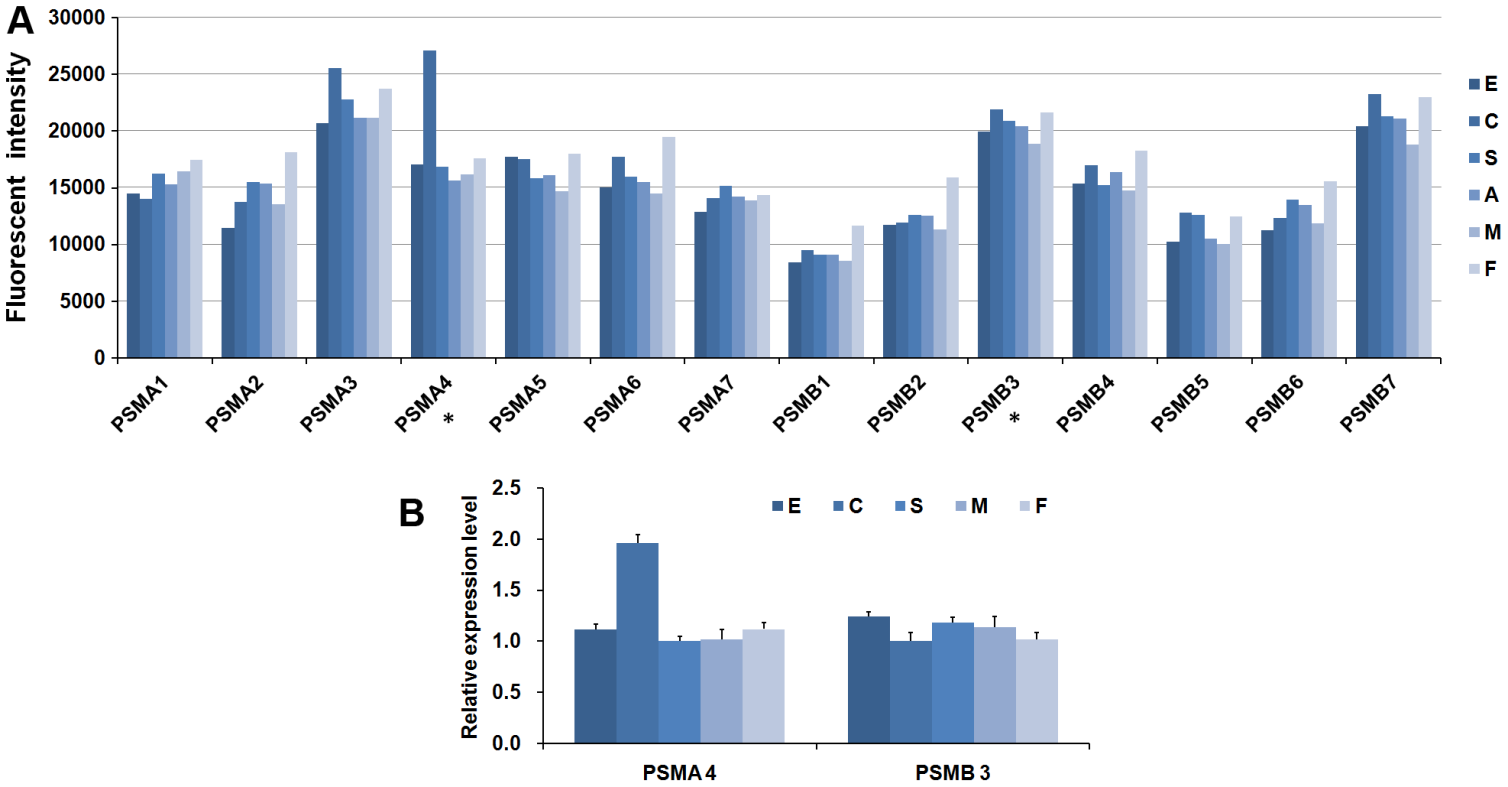
Expression profiles of the *S. japonicum* 20S proteasome in the four developmental stages. (A) The transcriptional profile of the *S. japonicum* 20S proteasome consisted of 14 subunits in the four developmental stages (E, eggs; C, cercariae; S, hepatic schistosomula; A, adult worm pairs; M, adult male worms; F, adult female worms). The data was obtained from our microarray dataset. The bar graphs represent the mean normalized fluorescent intensity values (Table S5) for the subunits. * Subunits selected for further validation by qRT-PCR. (B) The expression of two subunits of the *S. japonicum* 20S proteasome was validated by qRT-PCR analysis in the four developmental stages. The relative expression levels of genes were calculated using SDS v1.4 software (Applied Biosystems). The error bars represent standard deviation for three technical replicates.

We used an oligonucleotide microarray to measure the patterns of expression for the obtained proteases at four developmental stages in *S. japonicum*. After BLAST searching, 257 of 262 protease sequences were aligned with sequences used for our microarray design. Using a fluorescence signal value of 100 as the cut-off value, we ultimately extracted 247 protease sequences with probes that produced detectable signals in microarray analysis; the other 10 sequences maybe pseudogenes or expressed in other developmental stages or conditions (See supplementary Table S5 for all the protease gene expression data extracted from our microarray). Using fold change cut-off value ≥2 between any two developmental stages and *p*-value <0.05 (one tailed Student's *t*-test), a total of 136 differentially expressed protease genes were identified in the four developmental stages and 47 proteases in sexual distinction (Table S6). Hierarchal clustering was used to investigate the transcriptional patterns of proteases in the four developmental stages. The heat map showed that three major transcriptional patterns were closely related to the developmental stages: pattern I, genes were significantly up-regulated in the schistosomula and adult stages (the mammalian host dwelling schistosome life-stages); pattern II, genes were predominantly expressed in the egg stage; and pattern III, genes were highly expressed in the cercaria stage (Figure 3 and S2). Examples of protease genes with different expression patterns are presented in Table 2. Proteases with different expression patterns may be closely bound up with the host-parasite interaction. For instance, the *S. japonicum* elastase gene (SjCE, ACR27083.1/Sjp_0028090), which has been validated to be important for cercariae in host skin invasion [12], was clustered into expression pattern III (Table 2). After invading the mammalian host, the blood fluke's growth, development, and reproduction were dependent on the acquisition of nutrients from the host bloodstream [17]. Over the past few decades, an increasing number of proteases of different classes have been ascribed roles in host protein digestion in schistosomes (reviewed in references [9], [37]), including cysteine protease legumain [38], cathepsin B, C and L, aspartic protease cathepsin D, and metalloprotease leucine aminopeptidase. The majority of proteases mentioned above were clustered into expression pattern I (Table 2, Figure S2). The pair-wise comparative analysis of gene expression provided detailed information for the identification of proteases related to schistosome developmental biology and host-parasite interactions. In addition, a set of stably expressed proteases genes was extracted from the analyses. By setting 1.5% as the cut-off value for the coefficient of variation, 33 constantly expressed protease genes were identified, and the majority were abundantly transcribed among the four developmental stages or between adult males and females (Figure 4). These constantly expressed protease genes may play fundamental roles in the life cycle of blood flukes, but only the enzymes that are unique to the parasite may serve as potential targets for anti-schistosomal drugs. For example, the proteasome, multisubunit enzyme complex, plays a key role in non-lysosomal protein degradation. The *S. mansoni* proteasome subunit beta type 6 (Smp_034490) has been predicted to be a possible drug target using an in silico approach [39]. Several proteasome inhibitors have been developed and evaluated in clinical trials as anticancer drugs [40], which may be used to study on anti-parasite in future. In mammals, the 26S proteasome contains a barrel-shaped proteolytic core complex (the 20S proteasome) and two 19S regulatory cap subunits. The 20S proteasome is composed of a four-ring arrangement of alpha and beta subunits. Mammals have seven alpha and seven beta proteasome subunits, and all of the counterparts were identified in *S. japonicum* (Table S2) as the main part of threonine proteases. With the exception of proteasome subunit alpha 4, the subunits were found to be stably transcribed (Figure 5A). Using *S. japonicum* PSMD4 (one of 26S proteasome non-ATPase regulatory subunits) as a reference gene for qRT-PCR validation, the relative expression levels of two selected subunit genes correlated well with the microarray results (Figure 5B). The COP9 signalosome (CSN) is a conserved multiprotein complex typically consisting of eight subunits (CSN1–CSN8) that plays a crucial role in the ubiquitin-proteasome-mediated protein degradation pathway by regulating the activity of E3-cullin RING ubiquitin ligases (CRLs) [41]. As a multifunctional subunit in the CSN complex, CSN5 not only functions as the catalytic center for CSN isopeptidase activity, but also independently participates in important biological functions [42]. Two CSN subunit genes with metalloprotease motifs (CSN5, Sjp_0052560 and CSN6, Sjp_0109020) were also among the constantly expressed protease genes in *S. japonicum*. All of the results suggest that the schistosome proteases involved in the ubiquitin-proteasome pathway may be considered potential anti-schistosomal intervention targets in future research.

**Table 2:** Examples of protease genes with different transcriptional patterns.

Probe Set ID	Protein ID	Description	Family	Pattern
CNUS0000096031.1	CAX72323.1	cathepsin D	A1	I
Sja_S23875597	AAW24549.1	cathepsin D	A1	I
CNUS0000103808.1	P43157.1	cathepsin B, Antigen Sj31	C1	I
CNUS0000099486.1	AAC32040.1	cathepsin C	C1	I
CNUS0000105206.1	AAW25326.1	cathepsin L	C1	I
CNUS0000095508.1	P42665.1	legumain, antigen Sj32	C13	I
Sja_S53128320	AAG40738.1	leucine aminopeptidase	M17	I
Sja_S53128698	CAX69725.1	cathepsin A	S10	I
CNUS0000103418.1	Sjp_0085250	lysosomal Pro-Xaa carboxypeptidase	S28	I
Sja_S23873527	CAX71062.1	lysosomal Pro-Xaa carboxypeptidase	S28	I
CNUS0000096566.1	Sjp_0012570	calpain-11	C2	II
CNUS0000106349.1	CAX73243.1	leishmanolysin	M8	II
PG005389	AAX26409.2	leishmanolysin	M8	II
CNUS0000099717.1	AAX27148.2	leishmanolysin	M8	II
CNUS0000096440.1	Sjp_0011250	leishmanolysin	M8	II
Sja_S53119426	CAX75591.1	leishmanolysin	M8	II
Sja_S53119431	CAX75587.1	leishmanolysin	M8	II
CNUS0000096438.1	Sjp_0011230	leishmanolysin	M8	II
CNUS0000096528.1	Sjp_0012180	enterokinase	S1	II
CNUS0000097585.1	CAX73257.1	transmembrane protease, serine 6	S1	II
CNUS0000100039.1	Sjp_0049310	lysosomal aspartic protease precursor	A1	III
CNUS0000104565.1	Sjp_0097500	calpain	C2	III
Sja_S23873524	CAX73441.1	calpain-B	C2	III
Sja_S53122136	CAX73508.1	ubiquitin carboxyl-terminal hydrolase 2	C19	III
CNUS0000102855.1	Sjp_0079300	ubiquitin-specific peptidase 24	C19	III
CNUS0000099952.1	Sjp_0048370	leishmanolysin	M8	III
PG003490	Sjp_0067490	leishmanolysin	M8	III
PG003491	Sjp_0067500	leishmanolysin	M8	III
CNUS0000098041.1	ACR27083.1	elastase 2b	S1	III
CNUS0000096939.1	Sjp_0016520	prolyl oligopeptidase	S9	III

### Phylogenetic analysis and global expression profiling of the schistosome cathepsin gene families

**Figure 6:**
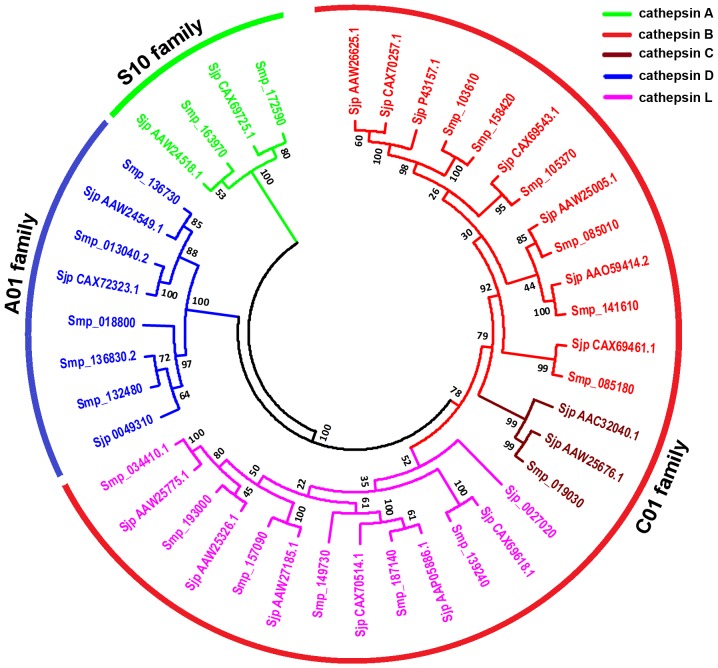
Molecular phylogenetic relationships between cathepsin family members from *S. japonicum* and *S. mansoni*. The unrooted phylogenetic tree was generated using MEGA 5.0 and the neighbor-joining method with 1000 bootstrap replicates. The bootstrap values are shown at the nodes. The tree was divided into three phylogenetic clusters designated as the cysteine protease C01 family, aspartic protease A01 family, and serine protease S10 family. Cathepsin A, B, C, D, and L were distinctly colored. Sjp, *S. japonicum* protein and Smp, *S. mansoni* protein.

Proteases frequently function not only as individual enzymes, but also in cascades or networks. A notable evolutionary switch occurred in one such protease network involved in protein digestion in the intestine [37]. In vertebrates, serine proteases of the trypsin family are mainly responsible for the work, whereas cysteine proteases of the papain family and aspartic proteases assume the role in invertebrates [9]. The cathepsins of blood flukes are thought to be the main proteases involved in the digestion of host blood proteins [37], [43]. The members of the cathepsin families characterized in *Schistosoma* species currently include cathepsin B (SjCB1, SmCB1, SjCB2, and SmCB2) [44]–[47], cathepsin C (SjCC and SmCC), cathepsin L (SjCL1/SjCF, SmCL1/SmCF, SmCL2, and SmCL3) [48], and cathepsin D (SjCD and SmCD). Although such a multienzyme network in invertebrate intestinal protein digestion has been validated using a combination of protease class-specific inhibitors and RNA interference in *S. mansoni*, the precise proteolytic cascade or network involving multiple proteases has not yet been determined definitively [49]. To identify the potential members of the schistosome cathepsin family, we used the above published schistosome cathepsin genes and *H. sapiens* cathepsin A gene (4CI9_A) as query sequences to perform a BLASTp search against our non-redundant degradome database. A total of 21 cathepsin genes were identified in the *S. japonicum* degradome and 20 genes in *S. mansoni*. The presence of cathepsin conserved domains (cd02620, cd02248, cd05490, cd05485, pfam00450 and pfam08773) was confirmed in all of the sequences using the conserved domain database on NCBI, and 18 *S. japonicum* cathepsins were predicted with signal peptides using the SignalP 4.1 server (Table S2). To examine the phylogenetic relationships among the cathepsin genes in the two *Schistosoma* species, we constructed a phylogenetic tree by aligning the 41 full-length schistosome cathepsin protein sequences using the neighbor-joining method in MEGA 5.0. The phylogenetic analysis showed that the schistosome cathepsin gene family can be divided into three classes of proteases (C01 family of cysteine proteases, A01 family of aspartic proteases, and S10 family of serine proteases) and five main kinds of cathepsins (cathepsin A, B, C, D, and L) (Figure 6).

**Figure 7:**
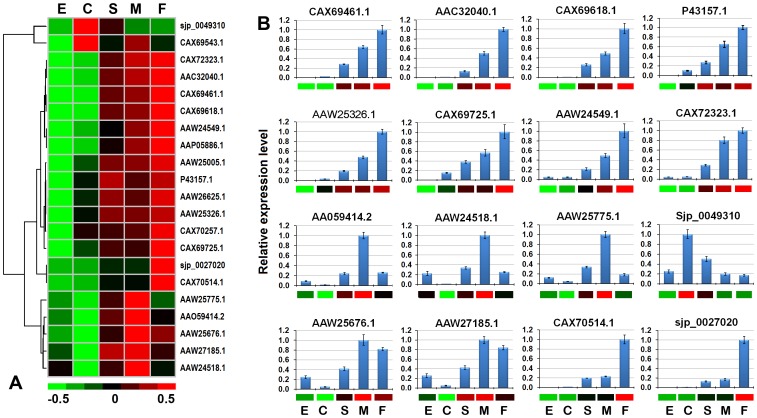
Expression profiles of the *S. japonicum* cathepsin family at four developmental stages. (A) The heat map shows the hierarchical clustering of 21 *S. japonicum* cathepsin genes in the four developmental stages (E, eggs; C, cercariae; S, hepatic schistosomula; M, adult male worms; F, adult female worms). The data were obtained from our microarray data. The color scale represents relative expression levels, with red as up-regulated and green as down-regulated. (B) The expression of 16 selected *S. japonicum* cathepsins at the four developmental stages was quantified by qRT-PCR analysis. The relative expression levels of genes were calculated using SDS v1.4 software (Applied Biosystems). The error bars represent the standard deviation for three technical replicates. The corresponding microarray gene expression data are presented below the bar graphs as heat maps, with up-regulated genes shown in red, down-regulated genes shown in green, and unchanged genes shown in black.

Next, we systematically analyzed the expression profile of the *S. japonicum* cathepsin family in the four developmental stages. As expected, the majority of these proteases were expressed primarily in schistosomula and adult worms, which is consistent with their roles in the digestion of host blood proteins (Figure 7A). The gene expression patterns detected by qRT-PCR for 16 selected *S. japonicum* cathepsin genes were generally consistent with the microarray results and could be further classified into several different expression patterns (Figure 7B). Eight of the 16 cathepsin genes were developmentally expressed from egg to adult worm (higher in adult female worms than adult male worms; Figure 7B). Among the eight cathepsin genes, schistosome cathepsin B1 (SjCB1, P43157.1 and SmCB1, P25792.1/Smp_103610), schistosome cathepsin C (SjCC, AAC32040.1 and SmCC, Q26563.1/Smp_019030), schistosome cathepsin L (SjCL2, AAW25326.1 and SmCL2, CAA83538.1/Smp_193000), and schistosome cathepsin D (SjCD, AAC37302/CAX72323.1 and SmCD, AAB63442/Smp_013040.2) have been shown to be important for host hemoglobin digestion [9], [37]. Notably, SjCD (CAX72323.1) was also highly expressed in eggs, in contrast to SjCB1, SjCC, and SjCL2, which were expressed at very low levels in eggs, if at all (Figure 7B, Table S5). Interestingly, we found that one cathepsin gene (Sjp_0049310) was highly expressed in the cercaria stage compared to the other three stages, two genes (Sjp_0027020 and CAX70514.1) were predominantly expressed in female worms, and three genes (AAW25775.1, AAO59414.2, and AAW24518.1) were strongly expressed in male worms (Figure 7B).

Why is there such a discrepancy in expression profiles among the schistosome cathepsins? The answer to this question will shed new light on the functions of parasite cathepsins, which is crucial for understanding parasite virulence and adaptation [43]. The different expression patterns of *S. japonicum* cathepsins among the developmental stages implied that schistosome cathepsins may be involved in diverse functions and biological processes. The cathepsin L protease family, as one of important protease families for parasites, has been extensively studied, especially in *Fasciola* species whose cathepsin L proteases have undergone a great expansion [43]. Herein, we constructed a phylogenetic tree using the protein sequences of schistosome cathepsin L and their homologous sequences characterized in other flatworms. The result showed that these proteases fell into two phylogenetic clades: three schistosome cathepsin Ls (cathepsin L1, L2 and L3) were allied closely in the first clade of the phylogenetic tree with other flatworm cathepsin Ls, and the others formed the second clade alone (Figure S3). The previous studies revealed that the cathepsin L proteases in the first clade were mainly presented in the excretory/secretory products of the adult worms, such as SmCL3 [48], FhCL1 and FhCL2 [50], [51], CsCL1 [52], and EmCL1 [53] which may contribute to the network of proteases involved in digestion of host proteins as nutrients. Notably, two *S. japonicum* cathepsin Ls (Sjp_0027020 and CAX70514.1) in the second clade were significantly up-regulated in adult females which may be related to reproduction.

Skin penetration, facilitated by cercarial secretions, is the initial event in infection of the mammalian hosts by *Schistosoma* species. Understanding the molecular and biochemical mechanisms of parasite invasion could provide a theoretical basis for rational vaccine and drug development. Previous studies from other groups revealed distinct invasion strategies among schistosome blood flukes [9], [54], [55]. In *S. mansoni*, cercarial elastases play essential roles in host skin invasion, and multiple elastases have been identified in genome-wide analyses [56]–[58]. However, *S. japonicum* may mainly utilize a papain-like cysteine protease to facilitate host invasion [58], [59]. Schistosome cathepsin B2 has been shown to degrade multiple host skin proteins, and *S. japonicum* has 40-fold greater cathepsin B activity in cercarial secretions than *S. mansoni* [59]. We found that *S. japonicum* cathepsin B2 (SjCB2, AAO55414.2) exhibits a distinct expression pattern compared to SjCB1 (P43157.1), which plays a key role in the digestion of host hemoglobin (Figure 7B). Although the relative expression of SjCB1 was the lowest in cercariae according to qRT-PCR analysis, the signal produced in the microarray analysis of cercariae was very strong (Table S5). SjCB1 and cathepsin B isoforms (AAW26625.1 and CAX70257.1) were also detected via strong hybridization signals in the microarray analysis of the cercaria stage (Table S5). These *S. japonicum* cathepsin B isoforms may be also involved in host skin invasion, in addition to their roles in host protein digestion. Defining the roles of these major enzymes will not only provide a clearer understanding of the functions of the complex parasite protease network, but also provide insights into which of these proteases are logical targets for the development of chemotherapy for parasitic diseases [49], [60].

### The schistosome stage- and gender-specific or predominantly expressed proteases

Notably, most attention has been focused on the schistosome aspartic and cysteine proteases that assist worms in obtaining nutrients from the host. Except the ‘cercarial elastase’ serine proteases, which facilitate host invasion by infective schistosome larvae, few serine proteases have been identified and characterized in the past few years [61]. Recently, two trypsin-like serine proteases of the S01 family (Smp_030350 and Smp_103680) were shown to be predominantly expressed in *S. mansoni* eggs [61], and their counterparts (CAX73257.1 and Sjp_0012180) in *S. japonicum* were found to have similar expression patterns in our research (Table 2, Figure S2). Remarkably, we found that several serine proteases have similar expression patterns as those involved in host protein digestion. For instance, *S. japonicum* cathepsin A (CAX69725.1, S10 family), also known as carboxypeptidase C, had a similar expression pattern as SjCB1 (P43157.1), SjCC (AAC32040.1), and SjCL2 (AAW25326.1) (Figure 7B). Two lysosomal Pro-Xaa carboxypeptidases (CAX71062.1 and Sjp_0085250, S28 family), which can hydrolyze carboxy-terminal amino acids linked to proline in peptides, had a similar expression pattern as SjCD (CAX72323.1 and AAW24549.1) (Table S5). It will be engrossing to determine whether these serine proteases are members of the multienzyme network involving in host protein digestion by schistosome parasite.

**Figure 8:**
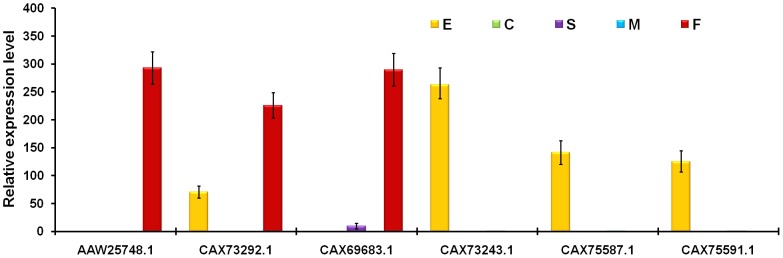
Expression analysis of six stage- and gender-specific or predominantly expressed genes using qRT-PCR. The expression was validated in the four developmental stages (E, eggs; C, cercariae; S, hepatic schistosomula; M, adult male worms; F, adult female worms) by qRT-PCR analysis. The relative expression levels of genes were calculated using SDS v1.4 software (Applied Biosystems). The error bars represent the standard deviation for three technical replicates.

In the complex lifecycle of schistosomes, the adult females pairing with adult males finally reside in the mesenteric or bladder circulation, where they produce infectious eggs. The majority of the eggs trapped in the host tissues, resulting in serious granulomatous reactions and fibrosis, are the major cause of pathology in schistosomiasis; the others eliminated into the environment with the host feces or urine are responsible for lifecycle progression [17]. As eggs play central roles in the pathology of schistosomiasis and transmission of the blood fluke, understanding the female reproductive biology and egg development could lead to novel strategies for combating schistosomiasis. Two *S. mansoni* tyrosinases specifically expressed in adult female worms have been shown to be critical for egg formation and production [62]. Three *S. japonicum* serine proteases (CAX69683.1, AAW25748.1, and CAX73292.1) were found to be specifically or abundantly expressed in adult female worms in microarray and qRT-PCR analyses (Figure 8, Table S5). The *S. japonicum* serine protease (CAX69683.1, S33 family) was annotated as putative lysosomal acid lipase (LAL) or cholesterol esterase. LAL plays a critical role in the hydrolysis of triglycerides and cholesterolesters, and LAL deficiency in humans leads to two phenotypes, cholesterolester storage disease and Wolman disease [63]. Fatty acid oxidation (FAO) is essential for schistosome egg production, which is consistent with the finding that fecund female worms possess abundant fat reserves, whereas virgin females have significantly lower lipid stores [64]. Meanwhile, genome-wide analysis of the metabolic pathway reveals that schistosomes can not *de novo* synthesize fatty acids or sterols, and the parasite genome certainly encodes multiple transporters and lipases to exploit fatty acids and cholesterol from the hosts [12]. As *S. japonicum* LAL (SjLAL, CAX69683.1) was significantly up-regulated in fecund female worms, and the expression pattern coincided with the previous FAO finding in schistosomes, SjLAL may be critical for female reproduction and the biological function of this protease needs to be investigated further.

The other two female-specific or highly expressed proteases (AAW25748.1 and CAX73292.1, S01 family) were annotated as trypsin-like serine proteases in *S. japonicum*. In the genetic model *Drosophila melanogaster*, female reproductive tract proteins play essential roles in sexual reproduction, and five mating-induced serine proteases expressed in the female reproductive tract have been identified using EST collections and microarray analyses [65]; the two *S.japonicum* trypsin-like serine proteases share the same conserved domain (cd00190) with these mating-induced serine proteases. Thus, the function of the two schistosome proteases may also be associated with sexual reproduction and could serve as new potential anti-schistosomal intervention targets.

As one of the most expanded gene families in schistosomes compared to their mammalian hosts, the M8 family of metalloproteases may yield new and valuable insights about the requirements for a parasitic lifestyle [12]. This family is composed of leishmanolysins (also called invadolysins), which were first reported in the protozoan parasite *Leishmania*. Fourteen putative M8 family members have been identified in the *S. japonicum* genome. This family includes important surface proteases of parasitic protozoa that play critical roles in the degradation of host extracellular matrix proteins to facilitate tissue or cell invasion [66]. The majority of leishmanolysins identified in our microarray were egg-enriched or cercaria-enriched genes (Table S5). Three egg-specific or predominantly expressed leishmanolysin genes (CAX73243.1, CAX75587.1, and CAX75591.1) were further validated by qRT-PCR (Figure 8). Analysis of *S. mansoni* cercarial secretions showed that leishmanolysin, now annotated in the genome as invadolysin, ranked second only to cercarial elastase as the most prominent component [67]. All of the cercaria-up-regulated leishmanolysins (Sjp_0048370, Sjp_0067490, and Sjp_0067500) (Table 2 and S5) have high homology (identity ≥65%) with *S. mansoni* invadolysins (Smp_153930, Smp_090100 and Smp_090110), which Parker-Manuel *et al* found to be significantly up-regulated in intramolluscan germ balls [68]. Numerous proteins utilized by the cercaria for host invasion have been suggested to be expressed during the development of germ balls in the snail [67]. Thus, it is tempting to speculate that leishmanolysin (invadolysin) may also contribute to tissue invasion by schistosome cercariae, besides cercarial elastase and cathepsin B. These egg-up-regulated leishmanolysins may also play vital roles in the release of eggs from host tissues or the hatching of miracidia from eggs. Therefore, leishmanolysin inhibition could serve as a novel intervention strategy for schistosomiasis. All of the suppositions need to be validated by experiments, which will contribute to the determination of protease functions and further improve the development of novel intervention strategies for schistosomiasis.

### Conclusion

The present study presents the most comprehensive analysis of degradomes in *Schistosoma* species to date. A total of 262 *S. japonicum* proteases were identified and the global expression profile at four developmental stages was obtained by microarray analysis. The proteases can be divided into four clusters according to the transcriptional pattern: proteases significantly up-regulated in schistosomula and adult stages, proteases highly expressed in the cercaria stage, proteases predominantly expressed in the egg stage, and proteases constantly expressed among the four developmental stages. Numerous potential anti-schistosomal targets were identified with the expression profile information, including cathepsin A, trypsin-like serine proteases, lysosomal Pro-Xaa carboxypeptidases, lysosomal acid lipase, leishmanolysins, and the 20S proteasome. Although the functions of schistosome proteases remain largely unknown, and many experiments are needed to determine their precise functions, our analysis of the *S. japonicum* degradome establishes a firm foundation for future research on the specific function(s) of individual proteases or protease families and may help refine anti-proteolytic strategies in blood flukes.

## Supporting Information

Figure S1 Primary sequence alignment of *S. japonicum* protease sequence AAW26282.1 with *S. mansoni* protease sequence Smp_061510.1 and Smp_155220.
(TIF)

Figure S2 Three clusters of protease genes with different expression patterns among four developmental stages (E, eggs; C, cercariae; S, hepatic schistosomula; A, adult worm pairs). I, genes significantly up-regulated in the schistosomula and adult stages; II, genes abundantly expressed in the egg stage; III, genes highly expressed in the cercaria stage. The color scale represents relative expression levels, with red as up-regulated, green as down-regulated, and black as unchanged.
(TIF)

Figure S3 Phylogenetic relationships between cathepsin L proteases of *Schistosoma* species, *Fasciola* species, *C. sinensis* and *Echinococcus* species. The unrooted phylogenetic tree was constructed using MEGA 5.0 and the neighbor-joining method with 1000 bootstrap replicates. The bootstrap values are shown at the nodes. SjCL, *S. japonicum* cathepsin L; SmCL, *S. mansoni* cathepsin L; FhCL, *Fasciola hepatica* cathepsin L; FgCL, *Fasciola gigantica* cathepsin L; EgCL, *Echinococcus granulosus* cathepsin L; EmCL, *Echinococcus multilocularis* cathepsin L; CsCL, *C. sinensis* cathepsin L. The color bars represent the relative expression levels of the *S. japonicum* cathepsin Ls in the four developmental stages, with red as up-regulated and green as down-regulated. E, eggs; C, cercariae; S, hepatic schistosomula; M, adult male worms; F, adult female worms.
(TIF)

Table S1 List of primers used for qRT-PCR analysis.
(XLSX)

Table S2 Comprehensive information of the *S. japonicum* degradome.
(XLSX)

Table S3 Comparison of numbers of proteases in *S. japonicum*, *S. mansoni*, *E. multilocularis*, *C. sinensis*, and *H. sapiens* by clan and family.
(XLSX)

Table S4 Blast2Go annotation details of *S. japonicum* protease sequences.
(XLSX)

Table S5 Normalized microarray data of the *S. japonicum* degradome in four developmental stages.
(XLSX)

Table S6 Differentially expressed protease genes identified by pair-wise comparative analysis of gene expression between any two developmental stages or genders.
(XLSX)
