# Supplementary figures and images for: Expression Profile of the Schistosoma japonicum Degradome Reveals Differential Protease Expression Patterns and Potential Anti-schistosomal Intervention Targets
Source: PLoS Comput Biol. 2014 Oct 2;10(10):e1003856. doi: 10.1371/journal.pcbi.1003856 (PMC4183426; doi:10.1371/journal.pcbi.1003856)

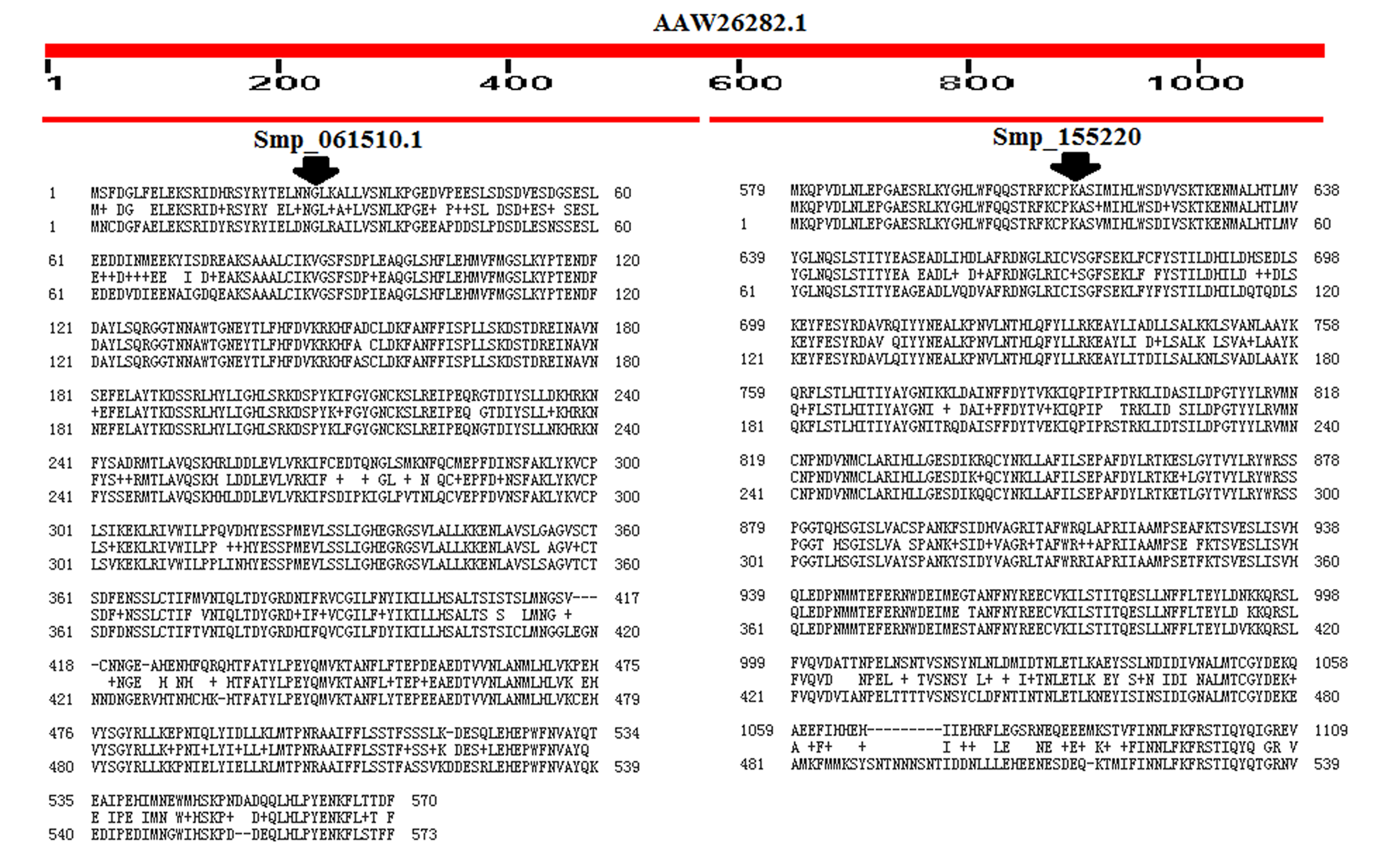

Supplement: Figure S1 — Primary sequence alignment of S. japonicum protease sequence AAW26282.1 with S. mansoni protease sequence Smp_061510.1 and Smp_155220. (TIF) [file pcbi.1003856.s001.tif]

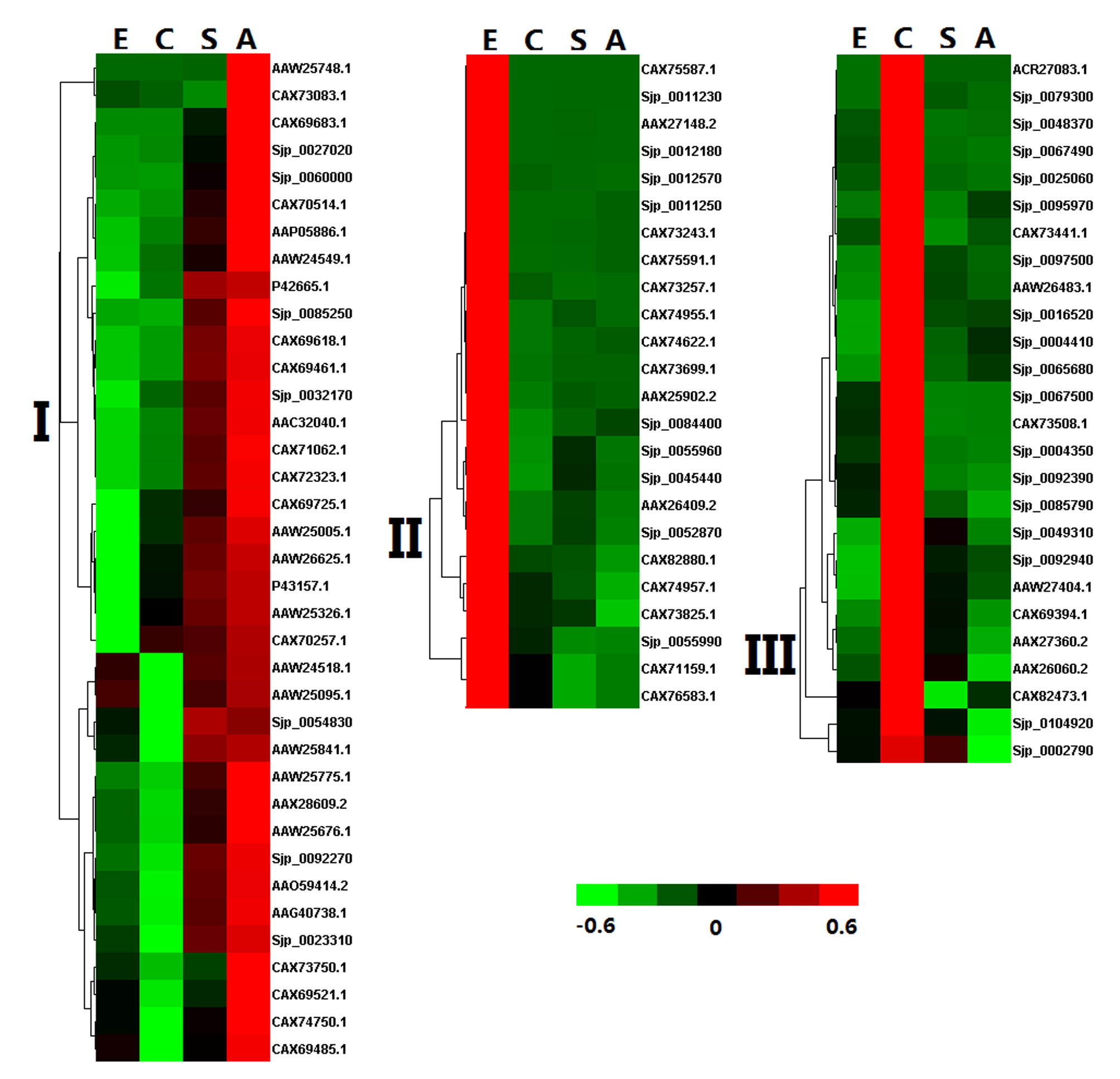

Supplement: Figure S2 — Three clusters of protease genes with different expression patterns among four developmental stages (E, eggs; C, cercariae; S, hepatic schistosomula; A, adult worm pairs). I, genes significantly up-regulated in the schistosomula and adult stages; II, genes abundantly expressed in the egg stage; III, genes highly expressed in the cercaria stage. The color scale represents relative expression levels, with red as up-regulated, green as down-regulated, and black as unchanged. (TIF) [file pcbi.1003856.s002.tif]

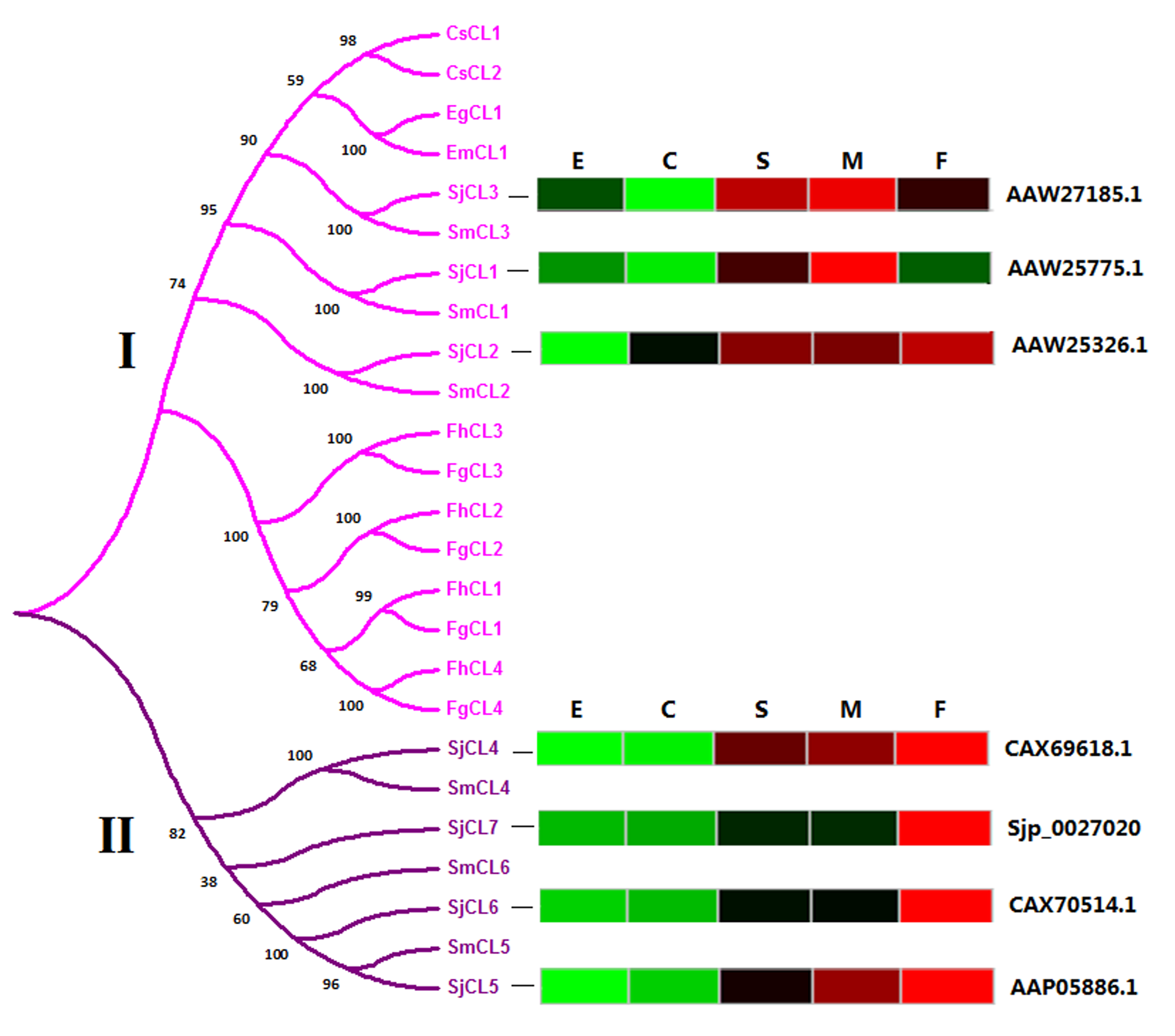

Supplement: Figure S3 — Phylogenetic relationships between cathepsin L proteases of Schistosoma species, Fasciola species, C. sinensis and Echinococcus species. The unrooted phylogenetic tree was constructed using MEGA 5.0 and the neighbor-joining method with 1000 bootstrap replicates. The bootstrap values are shown at the nodes. SjCL, S. japonicum cathepsin L; SmCL, S. mansoni cathepsin L; FhCL, Fasciola hepatica cathepsin L; FgCL, Fasciola gigantica cathepsin L; EgCL, Echinococcus granulosus cathepsin L; EmCL, Echinococcus multilocularis cathepsin L; CsCL, C. sinensis cathepsin L. The color bars represent the relative expression levels of the S. japonicum cathepsin Ls in the four developmental stages, with red as up-regulated and green as down-regulated. E, eggs; C, cercariae; S, hepatic schistosomula; M, adult male worms; F, adult female worms. (TIF) [file pcbi.1003856.s003.tif]
